# Role of prothrombin 19911 A>G polymorphism, blood group and male gender in patients with venous thromboembolism: Results of a German cohort study

**DOI:** 10.1007/s11239-020-02169-6

**Published:** 2020-06-27

**Authors:** Verena Limperger, Gili Kenet, Bettina Kiesau, Max Köther, Malin Schmeiser, Florian Langer, David Juhl, Maria Shneyder, Andre Franke, Ulrich K. Klostermeier, Rolf Mesters, Frank Rühle, Monika Stoll, Dagmar Steppat, Dorothee Kowalski, Angela Rocke, Piotr Kuta, Tido Bajorat, Antje Torge, Bruno Neuner, Ralf Junker, Ulrike Nowak-Göttl

**Affiliations:** 1 UKSH, Institute of Clinical Chemistry, Hemostasis Unit, Arnold-Heller-Str. 3 Building 17, Kiel, 24105 Germany; 2 National Hemophilia Center, Institute of Thrombosis and Hemostasis, Sheba Medical Centre, Tel-Hashomer, Israel; 3 The Amalia Biron Research Institute of Thrombosis & Hemostasis, Tel Aviv University, Tel Aviv, Israel; 4 Department of Hematology & Oncology, Univ. Hospital Hamburg, Hamburg, Germany; 5 Institute of Transfusion Medicine, Univ. Hospital Kiel & Lübeck, Lübeck, Germany; 6 Institute of Clinical Molecular Biology, Christian-Albrechts-University of Kiel, Kiel, Germany; 7 Department of Medicine/ Hematology & Oncology, Univ. Hospital Münster, Münster, Germany; 8 Institute of Human Genetics, Westfälische-Wilhelms-University, Münster, Germany; 9 Cardiovascular Research Institute Maastricht, Maastricht University, Maastricht, The Netherlands; 10 Department of Anesthesiology and Intensive Care Medicine, Charité – Universitätsmedizin Berlin, Campus Charité Mitte and Campus Virchow-Klinikum, Berlin, Germany

**Keywords:** Prothrombin 19911 A>G polymorphism, Thrombosis, Recurrence, Male gender

## Abstract

The role of the A>G polymorphism at position 19911 in the prothrombin gene (factor [F] 2 at rs3136516) as a risk factor for venous thromboembolism [VTE] is still unclear. To evaluate the presence of the F2 polymorphism in VTE patients compared to healthy blood donors and to adjust the results for common inherited thrombophilias [IT], age at onset and blood group [BG], and to calculate the risk of VTE recurrence. We investigated 1012 Caucasian patients with a diagnosis of VTE for the presence of the F2 rs3136516 polymorphism and compared these with 902 healthy blood donors. Odds ratios [OR] together with their 95% confidence intervals were calculated adjusted for F5 at rs6025, F2 at rs1799963, blood group, age and gender. In addition, we evaluated the risk of recurrent VTE during patient follow-up calculating hazard ratios [HR] together with their 95% CI. Compared with the AA wildtype, the F2 GG and AG genotypes (rs3136516) were associated with VTE (OR 1.48 and 1.45). The OR in F5 carriers compared to controls was 5.68 and 2.38 in patients with F2 (rs1799963). BG “non-O” was significantly more often diagnosed in patients compared to BG “O” (OR 2.74). VTE recurrence more often occurred in males (HR 2.3) and in carriers with combined thrombophilia (HR 2.11). Noteworthy, the rs3136516 polymorphism alone was not associated significantly with recurrence. In Caucasian patients with VTE the F2 GG/GA genotypes (rs3136516) were moderate risk factors for VTE. Recurrence was associated with male gender and combined thrombophilia.

**Electronic supplementary material:**

The online version of this article (10.1007/s11239-020-02169-6) contains supplementary material, which is available to authorized users.

## Introduction

Acquired risk factors such as surgery, immobilization, pregnancy, smoking, oral contraceptives and obesity, as well as inherited risk factors simultaneously contribute to the onset of venous thromboembolism [VTE] [1–3]. Rare inherited risk factors such as antithrombin-, protein C- and protein S deficiency are considered as high risk factors for VTE, whereas the factor [F] 5 [rs6025] and F2 [rs1799963] mutations are considered as more commonly diagnosed but being rather mild risk factors [1–5]. The A>G polymorphism at position 19911 of the prothrombin gene, e.g. F2 at rs3136516, located in intron 13 of the prothrombin gene is associated with increased plasma prothrombin levels, but its role as a risk factor for VTE is still unclear [6–11]. There is a small number of case control studies investigating the role of the F2 at rs3136516 polymorphism and its association with VTE. Of these, two reports showed a moderate risk for VTE [8, 9], whereas another study showed no statistically significance [10].

Therefore, the objectives of this study were to (i) evaluate the presence of the prothrombin 19911 A>G polymorphism in adolescent and younger adult Caucasian VTE in- and outpatients compared to healthy blood donors and to adjust the results obtained for common inherited thrombophilias [IT], age at onset and blood group [BG]. The second objective of this study was to (ii) calculate in the patient cohort the risk of VTE recurrence independently of gender, common mild ITs including the F2 at rs3136516 polymorphism, age at first VTE onset and blood group.

## Material and methods

### Study population

Consecutive patients with a first symptomatic VTE were recruited between December 2008 and 2018 whether or not prothrombotic risk factors were present. Screening of thrombophilia was performed in 1012 adolescent and adult patients [14 to < 60 years] and 902 healthy blood donors [18 to < 61 years] from the same catchment area.

### Inclusion and exclusion criteria

Corresponding to the following criteria patients were included into the study: (i) age from puberty status > Tanner 2 [12] to 60 years at first VTE onset, (ii) objectively confirmed thrombosis and re-thrombosis with established imaging procedures such as Duplex- or Doppler- ultrasonography, computed tomography or magnetic resonance tomography for venous thrombosis and spiral computed pulmonary angiography or lung perfusion scintigraphy for pulmonary embolism. Patients were excluded from the study when they suffer from (i) central line-associated VTE, VTE linked to malignancy, antiphospholipid syndrome, inherited antithrombin-, protein C- or protein S-deficiency or (ii) when they were lost to follow-up or did not abide by the follow-up visits.

### Study aims

The primary study objective was to determine the individual VTE risk of the F2 mutation at rs3136516 in Caucasian VTE patients [n = 1012] compared to healthy blood donors [n = 902] adjusted for (i) common ITs, e.g. F5 at rs6025 and F2 at rs1799963, age at onset and blood group [1–3, 13, 14]. The secondary study objective was to investigate the time to VTE recurrence after withdrawal of antithrombotic therapy, adjust for the above listed potential confounders.

### Clinical procedures

Following a first VTE onset (in- and outpatients) and during regular follow-up visits [3–6 months (adolescents), 9–12 months, 2 years (adults)] we evaluated and re-evaluated patient’s disease history and possible risk factors causing VTE, such as the use of oral contraceptives, pregnancy, obesity, immobilization, surgery and smoking. Clinical data collection also included laboratory test results, antithrombotic therapy, including adherence to anticoagulation and duration and family history of VTE. Adolescent and adult patients were treated independently from the underlying IT risk factors according to the latest antithrombotic VTE therapy guideline. (Online Supplement) [15–17].

Details on blood sample collection are depicted in detail in the online supplement.

### Laboratory analyses

With written or oral parental consent mutations in F2 at rs3136516, F5 at rs6025 and F2 at rs1799963 as well as circulating levels of antithrombin, coagulation inhibitors, d-dimer concentration as well as lupus anticoagulants and antiphospholipid antibodies were investigated with standard laboratory techniques at VTE onset and were repeated during routine follow up visits [12, 14]. Especially, the analyses of protein C and protein S were performed at least three months after the index event and/or withdrawal of vitamin-K-antagonists and deficiency states of antithrombin, protein C or protein S were confirmed if values repeatedly persisted below the age-related reference ranges [14]. Criteria for the hereditary nature of a hemostatic defect were its presence in at least one further first or second-degree family member and/or the identification of a causative gene mutation.

Single nucleotide polymorphism [SNP] Genotyping for F2 A19911G (LRG_551t1:c.1726-59G>A, rs3136516) was performed by TaqMan PCR. The assay includes two allele specific TaqMan probes containing distinct fluorescent dyes and highly specific PCR primers to detect the target (Assay ID: C__11661574_10, ThermoFisher Scientific, RRID:SCR_018060). ABI 7900HT Fast Real-Time PCR System was used for analysis. Of note, the specific snip applied (rs3136516) does not show a significant deviation from Hardy Weinberg equilibrium in the healthy cohort of blood donors (p = 0.55).

### Statistics

Statistical analyses were performed using the MedCalc^®^ software bvba (version 16.4.3, Ostend, Belgium, RRID:SCR_015044) and StatView 5 software packages (SAS Institute Inc., RRID:SCR_017411). Continuous variables were expressed as mean (± standard deviation) or median [minimum–maximum] values and categorical data were expressed as counts and percentages. The Wilcoxon–Mann–Whitney-U-test investigated differences in continuous non-normal distributed variables between two independent groups. The Chi-square-test was used to analyze differences in binary and categorical data between for the primary study aim Odds ratios [OR] and their corresponding 95% confidence intervals [CI] were calculated using an univariable logistic regression model comparing patients with healthy controls (blood donors). The homozygous GG genotype and the heterozygous AG were compared with the AA wildtype (F2 at rs3136516). In order to evaluate an independent contribution to the risk of VTE and to adjust for further potential confounders (common IT, age at onset, blood group) [1–3, 13, 14] these variables were entered into a backward multivariable logistic regression model. In addition, to test for possible statistical interactions between F5 rs6025 and F2 rs313616 genotypes a multiplicative scale was incorporated in the model. Per “a priori” definition this backward model included variables with a p value of ≤ 0.2 and, vice versa, removed variables from the model if p was > 0.21. For the secondary study objective, i.e. the time to recurrence, we calculated the probability of VTE-free survival as a function of time utilizing the method of Kaplan and Meier (univariable analysis). The log rank test was used to test for differences in recurrence-free survival between groups. On the basis of previous reports (data presented in the Online Suppl. Tables 1 and 2) minimum sample size calculation for the comparison of two proportions was performed assuming a type I error of 0.05 and a type II error of 0.20 (sampling comparison of proportions) [18]. Patients were withdrawn from the survival analysis (censored cases) either at death unrelated to VTE recurrence or at loss to follow-up using data of the last clinical follow-up visit. In order to evaluate an independent contribution to the risk of recurrent VTE (dichotomous variables: recurrence “yes” versus “no recurrence”) and to adjust for further potential confounders (gender, common IT, age at first VTE onset, blood group) the hazard ratio (HR) together with 95% confidence intervals (CI) were estimated from Cox’s proportional hazards model. To further test the relationship between independent and dependent variables, the likelihood ratio test was performed. To test the proportional hazard assumption, one of the prerequisites for applying the Log-rank test or the Cox regression model fit, we used the cox.zph procedure in the package ‘survival’ (v3.1.8; Therneau TM, 2019) in R 3.4.3 [19]. A p value > 0.05 indicates no violation of the proportional hazard assumption. The recurrence rates were calculated as the number of recurrent events per 100 person-years.

### Ethics

The underlying multicenter cohort study was approved by the medical ethics committee of the University of Münster & Kiel [B304/16], Germany and written informed consent was provided in all cases prior to study participation.

## Results

Demographic data and thrombotic locations of patients (n = 1012) and healthy controls (n = 902) are depicted in Table 1. Frequency distribution of single & combined thrombophilic risk factors are given in Table 2 and in the online supplement.

**Table 1:** VTE manifestations in patients are depicted

	Patients with VTE n = 1012	Healthy controls n = 902
Age	36.8 ± 10.9	37.8 ± 14
Female	696 (68.8%)	443 (49.1%)
VTE location at onset: n [%]		
VTE leg	688 [68.0]	–
VTE and PE	192 [19.0]	–
CSVT	61 [6.0]	–
Multiple	32 [3.2]	–
VTE and ATE	39 [3.9]	
Recurrent VTE location: n [%]		
VTE leg	122 [68.5]	–
VTE and PE	1 [0.6]	–
CSVT	4 [2.2]	–
PE	47 [26.4]	–
VTE and ATE	4 [2.2]	–

*ATE* arterial embolism of venous origin, *CSVT* cerebral sinus vein thrombosis, *NA* does not apply, *n* number, *PE* pulmonary embolism, *VTE* venous thromboembolism

**Table 2:** Frequency distribution of single & combined thrombophilic risk factors in patients and controls are summarized

	Patients with VTE n = 1012	Healthy controls n = 902
Thrombophilia RF	n [%]	n [%]
Single RF		
F2 rs3136516 GG	261/1012 [25.8%]	206/902 [22.8%]
F2 rs3136516 GA	518/1012 [51.2%]	452/902 [50.1%]
F2 rs3136516 AA	231/1012 [22.8%]	244/902 [27.1%]
F2 rs1799963 GA/AA	41/642 [6.4%]	24/902 [2.7%]
F5 rs605 GA/AA	275/1012 [27.2%]	50/902 [5.5%]
Combined RF		
F2 rs3136516 GG and F5 rs605 GA/AA	80/1012 [7.9%]	19/902 [2.1%]
F2 rs3136516 GG and rs1799963 GA/AA	Not detected	Not detected
F2 rs3136516 GA and F2 rs1799963 GA/AA	23/642 [3.6%]	11/902 [1.2%]

*F2* factor 2, *F5*: factor 5, *n* number, *RF* risk factor, *VTE* venous thromboembolism

### Primary study aim (univariable analysis: Table 3)

Compared with the AA wildtype the GG genotype (rs3136516) increases the risk of VTE with an OR of 1.39 (CI 1.04–1.73) and 1.21 (CI 0.97–1.51) in carriers of the AG variant. In addition, in the cohort investigated the OR of F5 carriers compared to healthy blood donors was 6.35 (CI 4.59–8.77) and 2.44 (1.47–4.05) in patients with F2 (rs1799963). Combinations between the homozygous F2 GG genotype at rs3136516 and F5 at rs6025 did not significantly influence the thrombotic risk when comparing patient with controls (OR 0.56; CI 0.29–1.09) and combinations between the factor 2 rs1799963 GA/AA variants and the homozygous GG (rs3136516) genotype were neither detected in cases nor in healthy controls, respectively. BG “non-O” was significantly more often found in patients with VTE compared to BG “O” (OR 3.77, CI 3.04–4.69). Furthermore, increasing age per year at first VTE onset enhances the VTE risk significantly by an OR of 1.01 (CI 1.02–1.04).

### Multivariable analysis (Table 3)

Backward logistic regression showed that compared with the AA wildtype the GG genotype (rs3136516) was independently associated with a diagnosis of VTE with an OR of 1.48 (95% CI 1.06–2.06) and with the OR of 1.45 (95% CI 1.1–1.92) in carriers in which the AG variant was genotyped. In addition, the OR of F5 in patients compared to healthy blood donors was 5.68 (95% CI 3.94–8.20) and 2.38 (95% CI 1.35–4.20) in patients with F2 (rs1799963). BG “non-O” was significantly more often found in patients with VTE compared to BG “O” (OR 2.74; 95% CI 2.11–3.55). Again, as depicted in univariable analysis increasing age per year at first onset retained its significance also in multivariate analysis (OR 1.03; 95% CI 1.02–1.04). Due to the “a priori” defined statistical cut-off p-values combinations between F5 rs6025 and F2 rs3135616 (multiplicative scale) were removed from the multivariable model and were not included in the final analysis.

**Table 3:** Uni- and multivariable analysis (logistic regression): odds ratios, 95% confidence intervals are depicted

Risk factor	Odds ratio (95% CI) Univariable analysis	Odds ratio (95% CI) Multivariable analysis
F2 rs3136516 AA	1	
F2 (rs3136516) GG	1.39 (1.04–1.73)	1.48 (1.06–2.06)
F2 (rs3136516) GA	1.21 (0.97–1.51)	1.45 (1.10–1.92)
F2 rs1799963 GG	1	
F2 (rs1799963) GA	2.44 (1.47–4.04)	2.38 (1.35–4.20)
F5 rs6025 GG	1	
F5 (rs6025) GA	6.35 (4.59–8.77)	5.68 (3.94–8.20)
Blood group 0	1	
Blood group non-0	3.77 (3.04–4.69)	2.74 (2.11–3.55)

*F2* factor 2, *F5* factor 5

### Secondary study aim

**Fig. 1:**
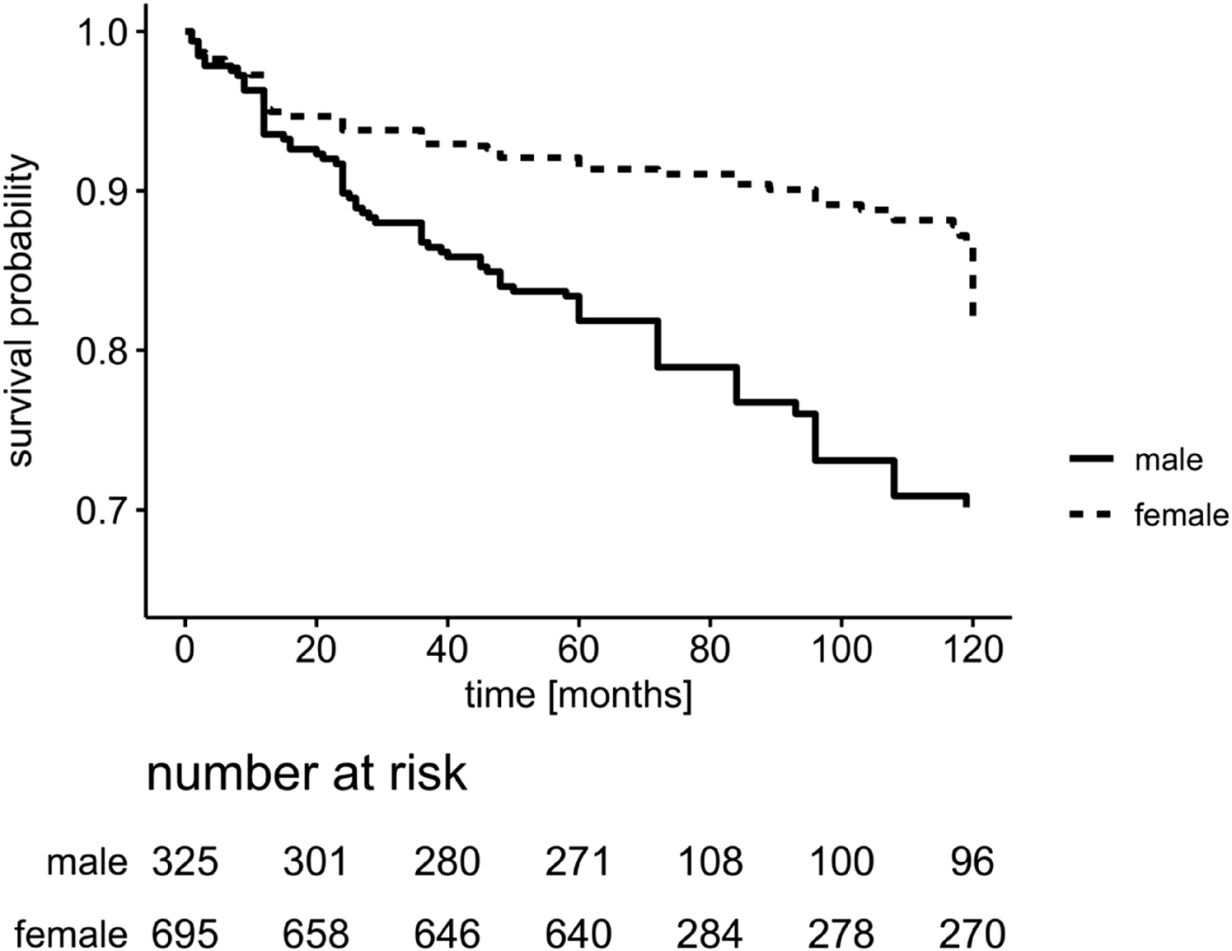
Probability of recurrence-free survival (y-axis) following a first symptomatic VTE onset is depicted: 13.1% of women compared to 26.5% of men developed a second thrombotic event (log rank value: p < 0.0001)

Within a mean time of 51.2 months (standard deviation: 43.5), recurrent VTE events (provoked 77%; locations depicted in Table 1) occurred in 178 out of 1012 patients (17.6%) following the first VTE onset (Fig. 1): 26.5% in men and 13.1% in women (p < 0.001). Gender associated incidence rates per 100 patient years (Table 4) were 4.3 (95% CI 3.4–5.3) in men and 1.9 (95% CI 1.6–2.4) in women (p < 0.0001). Further details of thrombotic locations and AC prior VTE recurrence are described in the online supplement material.

**Table 4:** Incidence rates of recurrent VTE per 100 patient years (gender and inherited thrombophilia) are depicted

Type of inherited thrombophilia	Exposure time (years)	Event (n)	Incidence per 100 PY % (95% CI)	*p* value
Gender: male	2014	87	4.3 (3.4–5.3)	< 0.0001
Gender: female	4690	91	1.9 (1.6–2.4)	
Any IT	3578	111	3.1 (2.6–3.7)	0.089
No IT	2860	67	2.4 (1.9–3.0)	
Combined IT	700	34	4.8 (3.4–6.8)	0.0006
Total	6704	178	2.6 (2.3–3.1)	

*CI* confidence interval, *PY* patient years

**Fig. 2:**
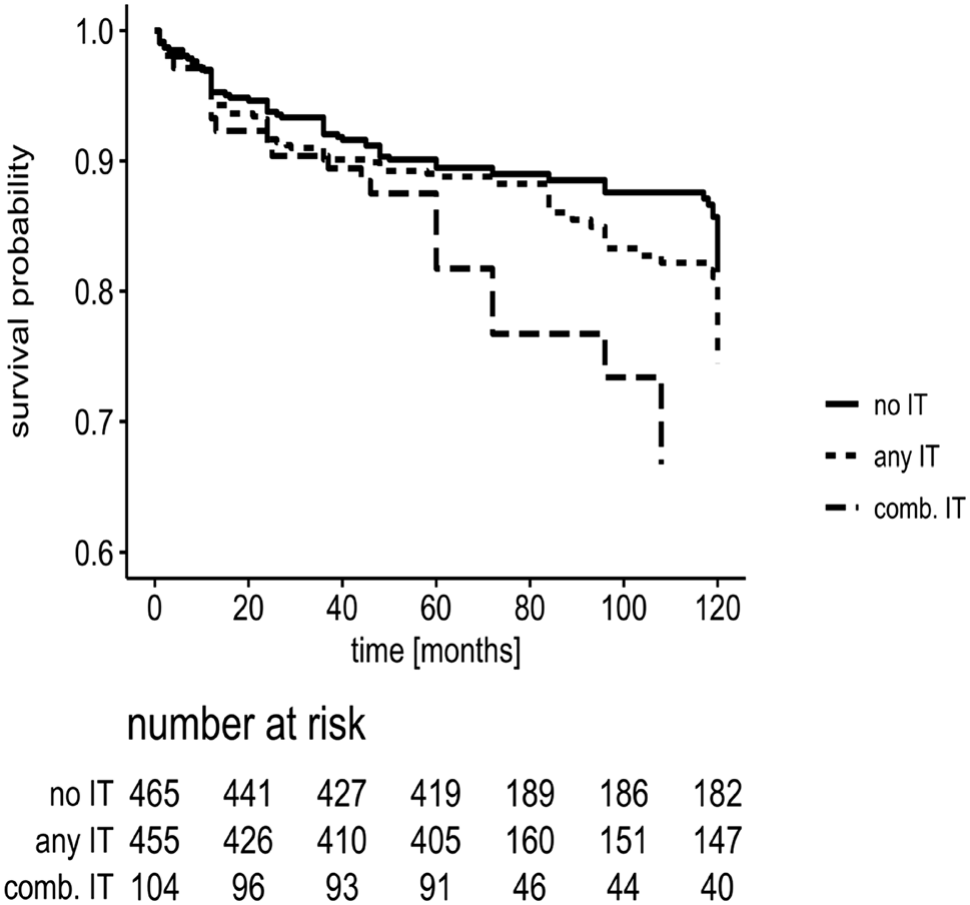
Probability of recurrence-free survival (y-axis) following a first symptomatic VTE onset is depicted: 32.7% of patients carrying combined thrombophilia variants compared to 15.6% without thrombophilia developed a second thrombotic event (log rank value: p < 0.0001). In addition, recurrence-free survival in patients with any thrombophilia is depicted

Whereas no significant associations were found between the risk of recurrent VTE and individuals classified as carriers of (i) the F2 mutation at rs3136516 “GG” subjects (OR 1.41; 95% CI 0.9–2.16) or in patients carrying (ii) the F2 rs179963 “GA” genotype (OR 1.81; 95% CI 0.87–3.76), results of the logistic regression model demonstrates that (iii) heterozygous F5 carriers had an increased OR of 1.86 (95% CI 1.22–2.83) to suffer from recurrent VTE with a further increase in patients additionally carrying the F2 GG/GA variant (rs3136516): OR 3.34 (95% CI 1.62–6.89). Patients with at least any IT compared to patients with no IT showed an increased odds ratio of 1.7 (95% CI 1.2–2.3) for a recurrence of VTE, while in patients with combined ITs the OR increased to 2.4 (95% CI 1.7–3.4). Of note, there was no significant association between the presence or absence of provoking risk factors and inherited thrombophilia (p = 0.93). The recurrence-free survival is depicted in Fig. 2: 32.7% of patients carrying combined ITs compared to 15.6% without IT developed a second thromboembolic event within 36 months (min–max: 1–120; log rank test: p < 0.0001). Multivariable analysis including time to recurrence demonstrates that male gender [hazard ratio (HR) 2.3; 95% CI 1.7–3.0] and combined ITs (HR 2.11; 95% CI 1.5–3.0) showed a significant impact on the risk of VTE recurrence. Noteworthy, in this specific data set, gender, F5, F2 rs3136516, F2 rs179963 and blood group did not violate the proportional hazard assumption. Remarkably, age, both as continuous term or grouped into three equal sized groups did violate the proportional hazard assumption and was thus not included in the multivariable analysis. Due to the “a priori” defined statistical cut-off p values the comparison between BG O and non-O was removed from the multivariate model. Incidence rates of recurrence per 100 patient years are depicted in detail in Table 4.

## Discussion

In the present longitudinal study in German patients with VTE we have shown that the presence of the F2 mutation at rs3136516 either in its GG or its GA genotype compared to the AA variant plays a moderate but independent role at first VTE onset with an adjusted odds ratio of 1.48 (GG genotype) and 1.45 (GA genotype), respectively. These findings were in agreement to study results described earlier in 2006 in an Italian cohort of patients (OR 1.5) and in line with data derived from the Netherlands with a 1.3-fold increased OR to suffer from VTE. As in our study, in the latter two cohorts the F2 rs3136516 GG genotypes were compared to AA carriers [8, 9]. The observation of a moderately enhanced association with VTE reported by us and by both cohorts published in 2006, however, is in contrast to findings reported by Perez-Ceballos et al. in 2002 and by Aradjanski et al. in 2014 [7, 10]. A summary of pooled data of previous reports [7–10] including our patients is depicted in the online supplement. Pooling results of overall four studies with 6150 patients with VTE and 6588 population-based controls, a modest increased association with VTE in carriers of the F2 (rs3136516) GG genotype of 1.3 (95% CI 1.2–1.4; p < 0.0001; heterogeneity [I^2^] 0.0%) was calculated. Interestingly, when investigating the role of this F2 intron polymorphism in a genome-wide association study the risk association with VTE was also modestly increased with an OR of 1.08 (1.06–1.11) [20].

In our cohort, combinations between the homozygous F2 GG genotype at rs3136516 and F5 at rs6025 additionally incorporated in the statistical model in a multiplicative scale did not significantly influence the thrombotic risk at VTE onset. This observation was in contrast to both cohort studies reported in 2006 [8, 9]. Combinations of the GG genotype with the F2 variant at rs1799963 GA/AA genotypes were not found, in accordance to previously reported data [8].

Of note, data of the multivariable analysis (primary study objective) confirmed findings by others that blood group “non-O” is an independent risk factor for a first VTE onset [21].

The contribution of male gender, age at first VTE onset and IT risk factors on VTE events were additionally investigated as secondary study aim in the patient follow-up study. During the patient-follow up after the first withdrawal of therapeutic AC we could demonstrate a significant risk increase of recurrent VTE in males (annual incidence rate of 4.3) compared to females (annual incidence rate of 1.9). Male gender remained an independent risk factor for recurrent VTE in multivariable analysis. The findings of our study, where VTE events were associated in the majority of cases with provoking risk factors, are in line with the higher recurrence risk in men following unprovoked VTE [22–24]. In contrast to findings from 2011 derived from a patient level meta-analysis in which the risk of recurrent VTE in men with a first provoked vascular occlusion was similar to that in women, the meta-analysis by McRae [23] found an enhanced risk for VTE recurrence in men. In our study the aforementioned increased risk of recurrence in men was neither affected by age at first VTE onset nor by underlying IT risk factors. Additionally, in the follow-up study, probably due to the small number of patients affected, the increased risk of blood group “non-O” and vice versa the protective role of BG O lost its significance in multivariable Cox regression analysis. Apart from possible explanations for the higher VTE recurrence in males compared to females discussed in more detail by Douketis [22], another explanation may be male gender per se: endogenous testosterone levels in males compared to females are clearly higher. Enhanced testosterone levels are associated with a hypo-fibrinolytic state possibly contributing to the onset of symptomatic thrombosis. The latter is not only described for endogenous elevated testosterone levels but also in patients receiving testosterone substitution therapy [25, 26].

As recently evaluated in a large international cohort of children and adolescents with VTE leading to thromboembolic and vascular stroke, the presence of more than one IT is an important risk factor for recurrent VTE [27]. Carriers of the F5 mutation showed a further risk increase in patients additionally carrying the F2 GG/GA variant (rs3136516) with the Odds ratio of 3.34 compared to 1.86 found in F5 carriers alone.

This study has several limitations: apart from (i) selection bias, i.e. age at onset, exclusion of subjects with central line- or malignancy-associated VTE, the non-enrollment of patients with antiphospholipid syndrome, limitations of the present study include (ii) the patient population ascertained with subjects of white race only. Thus, the results presented here cannot be directly transferred to other races, other age groups, to patients with VTE suffering from basic diseases not included in our cohort, such as malignancies. (iii) Continuously admitted patients were ascertained from 2008 onwards and were referred as in- and outpatients within a critical period in which improvement of imaging methods and clinical practice was noted [15, 16]. Results obtained at first thrombotic onset (case–control setting), however, are independent from change in imaging methods or clinical practice and the distribution of F5 rs605 GA/AA and F2 rs3136516 GA/AA is in line with previous reports: thus referral bias caused by inclusion of in- and outpatients seems to be unlikely. Since in- and outpatients were followed according to a similar study protocol a possible referral bias did not play a role with respect to recurrent VTE. It has to be mentioned here that the introduction and uptake of new therapeutic approaches such as DOACs might have influenced referral and treatment patterns. (iv) Although we observed a large number of patients with common mild IT risk factors this study might still be too small to show statistically significance: Although our findings in a clinical setting are in agreement with previously reported population based studies [8, 9], higher patient numbers are needed to rule out statistical errors. (v) Finally, we may have missed recurrent thrombosis in the patients investigated: whereas we may have overlooked asymptomatic VTE recurrence it is unlikely, however, that we would have missed symptomatic recurrences. This statement is underlined by the fact that the recurrence rates detected in this investigation are within the range reported by others [28, 29].

In conclusion in Caucasian adolescent and adult patients with a first VTE onset, the F2 GG/GA genotypes (rs3136516) contribute to the first onset of the disease. Findings of this study gave evidence that in patients with provoked VTEs (i) male gender and (ii) combined ITs, including the F2 mutation at rs3136516 may be regarded as important risk factors for VTE recurrence. These should be included in decision-making in regard to length of AC treatment. Whether (iii) blood group non-O should be included into a predictive scoring system, however, is part of an ongoing prospective study.

## Electronic supplementary material

Below is the link to the electronic supplementary material.

Supplementary file1 (DOCX 129 kb)
