# Correction to: Role of prothrombin 19,911 A > G polymorphism, blood group and male gender in patients with venous thromboembolism: results of a german cohort study

**DOI:** 10.1007/s11239-023-02809-7

**Published:** 2023-04-15

**Authors:** Verena Limperger, Gili Kenet, Bettina Kiesau, Max Köther, Malin Schmeiser, Florian Langer, David Juhl, Maria Shneyder, Andre Franke, Ulrich C. Klostermeier, Rolf Mesters, Frank Rühle, Monika Stoll, Dagmar Steppat, Dorothee Kowalski, Angela Rocke, Piotr Kuta, Tido Bajorat, Antje Torge, Bruno Neuner, Ralf Junker, Ulrike Nowak-Göttl

**Affiliations:** 1 UKSH, Institute of Clinical Chemistry, Hemostasis Unit, Arnold-Heller-Str. 3 Building 17, Kiel, 24105 Germany; 2 National Hemophilia Center, Institute of Thrombosis and Hemostasis, Sheba Medical Centre, Tel- Hashomer, Israel; 3 The Amalia Biron Research Institute of Thrombosis & Hemostasis, Tel Aviv University, Tel Aviv, Israel; 4 Department of Hematology & Oncology, Univ. Hospital Hamburg, Hamburg, Germany; 5 Institute of Transfusion Medicine, Univ. Hospital Kiel & Lübeck, Lübeck, Germany; 6 Institute of Clinical Molecular Biology, Christian-Albrechts-University of Kiel, Kiel, Germany; 7 Department of Medicine/ Hematology & Oncology, Univ. Hospital Münster, Münster, Germany; 8 Institute of Human Genetics, Westfälische-Wilhelms-University, Münster, Germany; 9 Cardiovascular Research Institute Maastricht, Maastricht University, Maastricht, The Netherlands; 10 Department of Anesthesiology and Intensive Care Medicine, Charité – Universitätsmedizin Berlin, Campus Charité Mitte and Campus Virchow-Klinikum, Berlin, Germany

**Journal of Thrombosis and Thrombolysis (2020) 51:494-501**

10.1007/s11239-020-02169-6

In the original version of the article:

1. The name of Ulrich K. Klostermeier has been misspelled; the correct author name is “Ulrich C. Klostermeier”

These have been corrected with this erratum.

