# Supplementary material for: Role of prothrombin 19911 A>G polymorphism, blood group and male gender in patients with venous thromboembolism: Results of a German cohort study
Source: J Thromb Thrombolysis. 2020 Jun 27;51(2):494–501. doi: 10.1007/s11239-020-02169-6 (PMC7886710; doi:10.1007/s11239-020-02169-6)
Supplement: Supplementary file 1 — Supplementary file1 (DOCX 129 kb) [file 11239_2020_2169_MOESM1_ESM.docx]

**Online supplement**

***Anticoagulant treatment:*** Based on standardized in-house[1, 2] treatment guidelines, patients with provoked VTE of the legs received anticoagulation (AC) between 3 and 6 months and the duration of AC in patients with unprovoked VTE and/or pulmonary embolism (PE) was prolonged up to 12 months. Prior to withdrawal of anticoagulation the D-dimer concentration was aimed to be settled below the aged-dependent cut-off values in two follow-up visits and had to stay within the normal ranges six weeks following AC removal. In addition, in patients with PE cardiovascular ECHO examination must be normal, e.g. without signs of left-ventricular dysfunction prior to AC removal.

***Blood sample collection:*** As previously described blood samples were collected from patients and controls at the study centers in the morning after a 12-hour fasting period. Samples were drawn by peripheral venipuncture into plastic tubes containing 1/10 by volume of 3.8% trisodium citrate (Sarstedt, Nümbrecht, Germany) and were immediately placed on melting ice. The blood samples from patients were collected 6-12 months after the acute thrombotic event, and at least 6 weeks after discontinuation of anticoagulation therapy. Platelet-poor plasma was prepared by double centrifugation at 3000 g at 4°C for 20 minutes, aliquoted in polystyrene long-term freezer storage tubes, stored at -70°C and thawed immediately before assay. DNA extraction was performed by a spin column procedure (Qiagen, Hilden, Germany) as previously described.

**Statistics:** Sample size calculations. [3]

**Table 1: Incidence and prevalence proportions according to known population-based data (reference 1-3). Minimum sample size calculated in male and female patients are depicted.**

| **Cohorts** | **Proportion of**  **F5 rs605 GA/AA** | **Proportion of**  **F2 rs3136516 GA/AA** | **Proportion of**  **F2 rs3136516GG**  **& F5 rs605 GA/AA** |
| --- | --- | --- | --- |
| VTE (%) | 12 | 5 | 29 (3) |
| Controls (%) | 5 (reference 1) | 2.5 (reference 2) | 23 (reference 3) |
| Cases (n) VTE | 276 | 984 | 871 |
| Controls (n) | 276 | 984 | 871 |

Abbreviations: F: factor; VTE: venous thromboembolism

**Table 2: Proportion of gender in recurrent deep vein thrombosis. Minimum sample size calculated in male and female patients are depicted.**

| **Cohorts** | **Proportion of**  **recurrence**  **(reference 4)** | **Proportion of**  **recurrence**  **(reference 5)** | **Proportion of**  **recurrence**  **(own data)** |
| --- | --- | --- | --- |
| male (%) | 20 | 30 | 27 |
| female (%) | 9 | 17 | 13 |
| cases (n)  male | 276 | 277 | 218 |
| cases (n) female | 138 | 139 | 109 |

**References tables:**

1. Li X, Cui L, Li Y, et al (2018) Prevalence and geographical variation of Factor V Leiden in patients with cerebral venous thrombosis: A meta-analysis. PLoS One 13:1–15. https://doi.org/10.1371/journal.pone.0203309

2. Gonzalez J V., Barboza AG, Vazquez FJ, Gándara E (2016) Prevalence and Geographical Variation of Prothrombin G20210A Mutation in Patients with Cerebral Vein Thrombosis: A Systematic Review and Meta-Analysis. PLoS One 11:e0151607. https://doi.org/10.1371/journal.pone.0151607

3. Martinelli I, Battaglioli T, Tosetto A, et al (2006) Prothrombin A19911G polymorphism and the risk of venous thromboembolism. J Thromb Haemost 4:2582–2586. https://doi.org/10.1111/j.1538-7836.2006.02216.x

4. Douketis J, Tosetto A, Marcucci M, et al (2011) Risk of recurrence after venous thromboembolism in men and women: patient level meta-analysis. BMJ 342:d813. https://doi.org/10.1136/bmj.d813

5. Kearon C, Parpia S, Spencer FA, et al (2019) Long-term risk of recurrence in patients with a first unprovoked venous thromboembolism managed according to d-dimer results; A cohort study. J Thromb Haemost 17:1144–1152. https://doi.org/10.1111/jth.14458

**Results:**

***Provoking risk factors at first VTE:*** 78.0% of our patients had provoked VTEs (n=789). Provoking risk factors were medical underlying diseases in 113 cases (such as cardiac, hepatic, infectious, metabolic or renal) and 293 index cases suffered from VTE after prolonged immobilization (> 3 days). Obesity (BMI > 27) was documented in 140 cases and 55 female patients were on oral contraceptives at the time of VTE onset. Smoking of more than 10 cigarettes per day was stated in 143 patients and non-classified provoking risks factors prior to VTE occurred in 228 patients. Combinations of risk factors were possible.

***VTE recurrence (secondary study aim):*** At VTE recurrence 77% of subjects had in similarity to the first VTE at least on provoking risk factor. Of note, 13 of 178 (7.3%) patients with a first VTE and withdrawal of anticoagulant therapy developed a second VTE under prophylactic low-dose LMWH (n=7), sub-therapeutic intake of vitamin-K-antagonists (n=4) or oral anti-factor Xa inhibitors (n=2), administered for secondary AC in recurring risk situations. In addition, prior to re-thrombosis none of the male or female patients affected had increased d-dimer concentrations.

***Meta-analysis (pooled data):*** In addition, according to previously published methodologies we have added our data to studies aforementioned [4–6] and have recalculated the risk to contribute to a first VTE onset as pooled ORs (meta-analysis) on the basis of observational studies. [7, 8] For the F2 at rs3136516 genotype total ORs (fixed & random effects) are depicted in a forest plot including study heterogeneity (I^2^).

Meta-analysis derived from data reported so far with respect to the GG genotype, F2 mutation at rs3136516, [4–6] are depicted in online figure 2: Without significant heterogeneity [I^2^=0%] the pooled OR (fixed and random effects model) derived from univariable individual calculations included 6150 patients with VTE and 6588 population-based controls and revealed a modest risk increase in carriers of the GG genotype of 1.3 (1.2-1.4).

**References:**

1. Kearon C, Akl EA, Ornelas J, et al (2016) Antithrombotic Therapy for VTE Disease. Chest 149:315–352. https://doi.org/10.1016/j.chest.2015.11.026

2. Monagle P, Chan AKC, Goldenberg NA, et al (2012) Antithrombotic therapy in neonates and children: Antithrombotic Therapy and Prevention of Thrombosis, 9th ed: American College of Chest Physicians Evidence-Based Clinical Practice Guidelines. Chest 141:e737S--801S. https://doi.org/10.1378/chest.11-2308

3. Machin D, Campbell MJ, Tan SB, Tan SH (2008) Sample Size Tables for Clinical Studies. Wiley-Blackwell, Oxford, UK

4. Pérez-Ceballos E, Corral J, Alberca I, et al (2002) Prothrombin A19911G and G20210A polymorphisms’ role in thrombosis. Br J Haematol 118:610–614. https://doi.org/10.1046/j.1365-2141.2002.03624.x

5. Martinelli I, Battaglioli T, Tosetto A, et al (2006) Prothrombin A19911G polymorphism and the risk of venous thromboembolism. J Thromb Haemost 4:2582–2586. https://doi.org/10.1111/j.1538-7836.2006.02216.x

6. Chinthammitr Y, Vos HL, Rosendaal FR, Doggen CJM (2006) The association of prothrombin A19911G polymorphism with plasma prothrombin activity and venous thrombosis: results of the MEGA study, a large population-based case-control study. J Thromb Haemost 4:2587–2592. https://doi.org/10.1111/j.1538-7836.2006.02257.x

7. Franchini M, Mannucci PM (2014) ABO blood group and thrombotic vascular disease. Thromb Haemost 112:1103–1109. https://doi.org/10.1160/th14-05-0457

8. Douketis J, Tosetto A, Marcucci M, et al (2011) Risk of recurrence after venous thromboembolism in men and women: patient level meta-analysis. BMJ 342:d813. https://doi.org/10.1136/bmj.d813

**Figure 1 (online supplement): Pooled OR (fixed and random effects model) derived from univariate individual calculations included 6150 patients with VTE and 6588 population-based controls are depicted. Horizontal lines correspond to the 95% CI intervals for each single study. The diamond and broke vertical line represent the summary estimate. The unbroken vertical line is at the null value (1) of the odds ratio and is equivalent to no difference.**

**
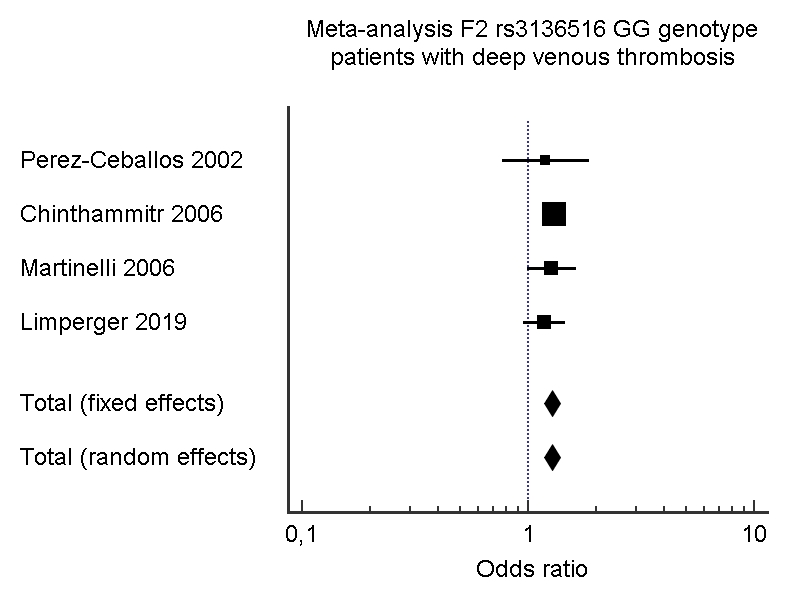
**
